# Thoracoscopic Lobectomy versus Open Lobectomy in Stage I Non-Small Cell Lung Cancer: A Meta-Analysis

**DOI:** 10.1371/journal.pone.0082366

**Published:** 2013-12-31

**Authors:** Yi-xin Cai, Xiang-ning Fu, Qin-zi Xu, Wei Sun, Ni Zhang

**Affiliations:** Department of Thoracic Surgery, Tongji Hospital, Huazhong University of Science and Technology, Wuhan, China; Roswell Park Cancer Institute, United States of America

## Abstract

The objective of the present meta-analysis was to evaluate the survival, recurrence rate, and complications in patients with stage I non-small cell lung cancer (NSCLC) who received video-assisted thoracoscopic surgery (VATS) or open lobectomy. A literature search was conducted on June 31, 2012 using combinations of the search terms video-assisted thoracic surgery, open thoracotomy, lobectomy, and non-small-cell lung cancer (NSCLC). Inclusion criteria were: 1) Compared video-assisted thoracic surgery (VATS) lobectomy with open lobectomy. 2) Stage I NSCLC. 2) No previous treatment for lung cancer. 4) Outcome data included 5-year survival rate, complication, and recurrence rate. Tests of heterogeneity, sensitivity, and publication bias were performed. A total of 23 studies (21 retrospective and 2 prospective) met the inclusion criteria. VATS was associated with a longer 5-year survival (odds ratio [OR] = 1.622, 95% confidence interval [CI] 1.272 to 2.069; P<0.001), higher local recurrence rate (OR = 2.152, 95% CI 1.349 to 3.434; P = 0.001), similar distant recurrence rate (OR = 0.91, 95% CI 0.33 to 2.48; P = 0.8560), and lower total complication rate (OR = 0.45, 95% CI 0.24 to 0.84; P = 0.013) compared to open lobectomy. VATS was also associated with lower rates arrhythmias, prolonged air leakage, and pneumonia but it did not show any statistical significance. Patients with stage I NSCLC undergoing VATS lobectomy had longer survival and fewer complications than those who received open lobectomy.

## Introduction

Since the introduction of thoracoscopic surgery, video-assisted thoracoscopic surgery (VATS) has become a viable option for the treatment of early stage lung cancer. Since the initial description of VATS in 1992, the number of VATS procedures for early stage lung cancer has steadily increased [1]. The past decade has seen an increase in the use of VATS for early stage lung cancer, and a recent report based on data from the Society of Thoracic Surgeons database indicated that VATS is used for 32% of all lobectomies in the United States [2]. VATS lobectomy has been shown to be associated with less postoperative pain, less surgical morbidity, fewer complications, and shorter hospitalization [3]–[9]. However, there is still much debate with respect to the role of VATS in lobectomy for the treatment of lung cancer. Though the feasibility and the safety of VATS for the treatment of early stage lung cancer has been proven [10], [11], there are persisting doubts regarding its oncological value; i.e., the potential compromise of oncological principles during surgery.

The objective of the present meta-analysis was to evaluate the survival, recurrence rate, and complications in patients with stage I non-small cell lung cancer (NSCLC) who received VATS or open lobectomy.

## Methods

### Literature Search Strategy

A search was conducted of PubMed, EMBASE, Google Scholar, and the Gray Journal including annual meetings of the American Society of Clinical Oncology and the American Society of Thoracic Surgery (chest surgery) using combinations of the search terms: video-assisted thoracic surgery, open thoracotomy, lobectomy, and non-small-cell lung cancer (NSCLC). The search date was June 31, 2012. Each publication was carefully examined, including the names of all authors, to avoid duplication of data.

### Selection Criteria

Studies were selected for inclusion in this analysis based on the following criteria. 1) Compared video-assisted thoracic surgery (VATS) lobectomy with open lobectomy. 2) Disease was non-small-cell lung cancer. 3) Stage I disease; no lymph node or distant metastasis. 4) No previous treatment for lung cancer. 5) Outcome data included 5-year survival rate, complication, and recurrence rate. Exclusion criteria for this analysis were as follows. 1) Abstracts, letters, editorials, and expert opinions, reviews without original data, case reports, and studies lacking control groups. 2) Studies concerned with unresectable lung cancer or recurrence after lobectomy. 3) Studies with no clearly reported outcomes of interest.

### Data extraction

Two independent reviewers extracted the data from eligible studies. A third reviewer was consulted for resolution of any disagreement. Data extracted included survival rates, recurrence rates, operative time, patent gender and age, disease stage, length of hospitalization, perioperative mortality, and complications including air leakage, arrhythmias, and pneumonia. The primary outcome measure was 5-year survival rate. Secondary outcomes were local and systematic recurrence rates, complications, and operation time.

### Data analysis

The 5-yr survival rate was used to evaluate treatment efficacy. The operation times, local recurrence, distant recurrence, total complications, prolonged air leakage, arrhythmia, and pneumonia were considered for safety evaluation. Proportion (%) or mean with standard deviations (SD) were summarized for the outcomes, and were compared between participants who received VATS or open lobectomy. Any χ^2^-based test of homogeneity was performed using Cochran's Q statistic and calculated I^2^, the percentage of the total variability in effect estimates among trials that is due to heterogeneity rather than chance. If the I^2^ statistic (>50%) indicated heterogeneity existed between studies, a random-effects model was calculated. Otherwise, fixed-effects models were used. Combined summary statistics of the odds ratios (ORs) or mean difference for individual studies were shown. All statistical assessments were 2-sided, and a P value<0.05 was considered to indicate statistical significance. Moreover, sensitivity and publication bias analysis were applied for the primary outcome, i.e., the 5-year survival rate. Sensitivity analysis was performed based on the leave-one-out approach. A Funnel plot and the fail-safe N (which indicates whether the observed significance is spurious or not) were used to assess possible publication bias. All analyses were performed using Comprehensive Meta-Analysis statistical software, version 2.0 (Biostat, Englewood, NJ, USA).

## Results

### Literature search

A total of 23 studies (21 retrospective and 2 prospective) met the inclusion criteria, and were included in this study. Briefly, 630 records were identified by the database searches and screened for relevance. After excluding non-relevant studies (n = 583) and duplicates (n = 17), 30 full text articles were assessed for eligibility. Of these 30 studies, the disease type was not specified in 2, and other stage disease was also included in 5. These 7 studies were excluded, and thus 23 were included in this meta-analysis. The included studies are listed in Table 1. Of note, data of the outcomes specified for the current analysis were not included in each of the 23 studies.

**Table 1:** Studies included in the meta-analysis.

				Open Lobectomy				VATS			
Study Design	Reference Number	1st Author (year)	Disease Stage	N of patients	% male	Age, y	Operation Time, min	N of patients	% male	Age, y	Operation Time, min
Retrospective	[12]	Park JS (2011)	pIA+IB	136	51.5	60	ND	136	51.5	60	ND
Retrospective	[13]	Ilonen IK (2011)	cIA+IB	212	59.4	65	ND	116	50	69	ND
Retrospective	[14]	Kawachi R (2009)	cIA+IB	176	65.9	67	ND	73	42.5	65	ND
Retrospective	[15]	Sakuraba M (2007)	cIA	56	58.9	63	ND	84	60.7	66	ND
Retrospective	[16]	Whitson BA (2007)	cIA+IB	88	48.9	65	ND	59	49.1	67	ND
Retrospective	[17]	Park BJ (2007)	cIA+IB	122	31.1	67	ND	122	31.1	67	ND
Retrospective	[18]	Tashima T (2005)	cIA+IB	173	44.5	67	185±42	67	37.3	68	175±66
Retrospective	[19]	Inada K (2000)	cIA+IB	30	73.3	67	222±17.7	24	70.8	67	280±19.7
Retrospective	[20]	Ohbuchi T (1998)	cIA	35	57.1	59	195.1±38.4	35	54.3	61	216.6±48.2
Retrospective	[21]	Flores RM (2009)	cIA	343	34.1	67	ND	398	38.2	67	ND
Retrospective	[22]	Shigemura N (2006)	cIA	55	52.7	62	159±28	81	49.4	ND	216.9±40.67
Retrospective	[23]	Muraoka M (2006)	cIA	42	73.8	65	293±83	43	53.5	65	288±66
Retrospective	[24]	Shiraishi T (2006)	cIA	79	65.8	66	224.8±64.5	81	59.3	63	226.7±48.9
Retrospective	[25]	Sawada S (2007)	cIA+IB	123	46.1	65	ND	165	63.4	65	ND
Retrospective	[26]	Tatsumi A (2003)	cIA+IB	121	62.8	68	230±57.6	118	56.8	66	219±61.67
Retrospective	[26]	Yang X (2009)	pIA+IB	98	ND	52	ND	43	ND	54	ND
Retrospective	[28]	Koizumi K (2002)	cIA	9	ND	71	ND	25	ND	74	ND
Retrospective	[29]	Sugiura H (1999)	cIA+IB	22	27.3	61	196±64	22	45.5	62	227±47
Retrospective	[30]	Nomori H (2001)	cIA+IB	22	50	64	270±84.9	22	50	64	281±81
Retrospective	[31]	Yim AP (2000)	cIA+IB	18	55.6	58	82±27	18	66.7	63	78±36
Retrospective	[32]	Tajiri M (2007)	cIA+IB	61	67.2	64	254.4±65.1	231	58.4	ND	258.6±51.28
Prospective	[33]	Sugi K (2000)	cIA	52	55.8	65	ND	48	58.3	66	ND
Prospective	[34]	Kirby TJ (1995)	cIA+IB	31	45.2	62	175±93	30	40	58	161±61

ND, not derived; VATS, video-assisted thoracoscopic surgery.

### Primary outcome (5-year survival rate)

**Figure 1:**
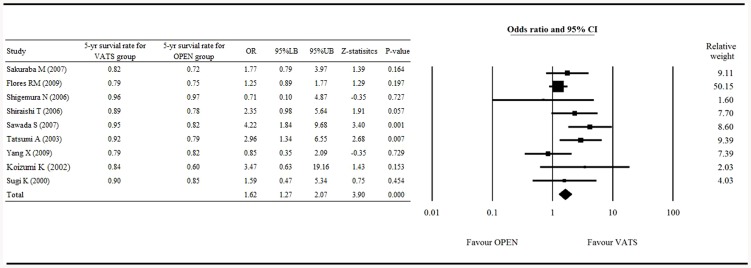
Forest plot of the 5-year survival rates of the VATS vs. open surgery groups. OR, odd ratio; LB, lower boundary; UB, upper boundary; CI, confidence interval.

The Forrest plot of the 5-year survival rate between patients who received VATS and those who received open surgery is shown in Figure 1. Nine studies [15], [21], [22]–[27], [29], [33] with complete survival rate data were included in the analysis. The heterogeneity test showed a fixed effect model was considered with a Q statistic = 13.652, and I^2^ = 41.401 (P = 0.091). The result, which showed an overall OR = 1.622 (95% confidence interval [CI] 1.272 to 2.069), significantly favored VATS over open surgery with respect to 5-year survival, with a Z-statistic = 3.898 (P<0.001; Fig. 1).

### Sensitivity analysis

**Figure 2:**
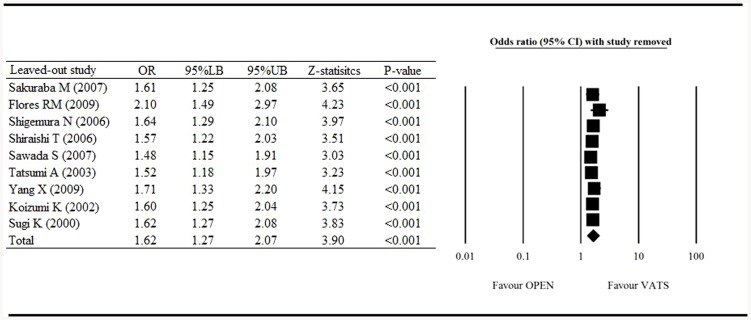
Sensitivity analysis of the influence of individual studies on pooled estimate for 5-year survival rate. OR, odd ratio; LB, lower boundary; UB, upper boundary; CI, confidence interval.

Figure 2 shows the results of the meta-analysis of the 5-year survival rate, with one study removed in turn. The results indicated that even with each of the studies removed in turn, the direction and magnitude of combined estimates did not have a large variation. This result indicates that the meta-analysis was proven to have good reliability.

### Publication bias

**Figure 3:**
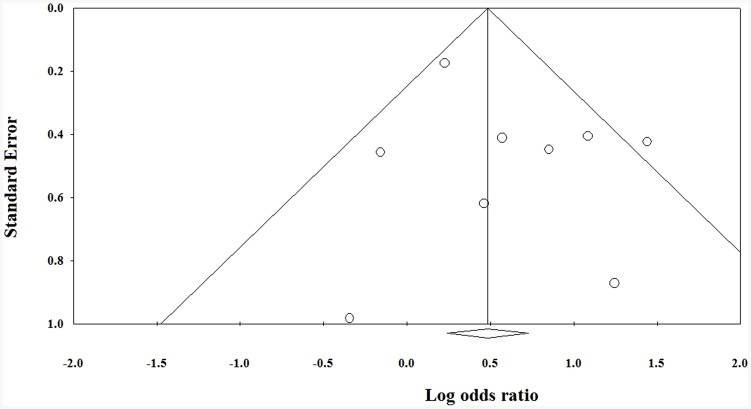
Funnel plot of the standard error by mean difference for 5-year survival rate.

The Funnel plot for publication bias (standard error by 5-year survival rate) demonstrated marked evidence of symmetry (Fig. 3), indicating a publication bias did not exist. The combined effect size yielded a Z value of 4.051, with a corresponding P value<0.001. This result indicates that the fail-safe N value was relevant.

### Secondary outcomes (local recurrence rate, distant recurrence rate)

**Figure 4:**
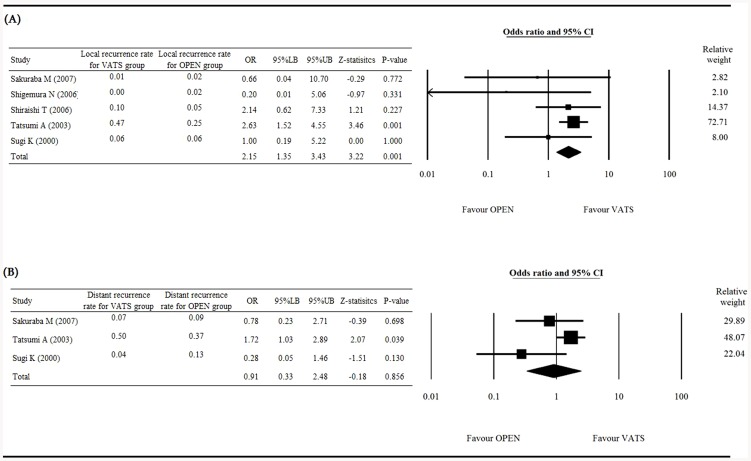
Forest plot for the VATS vs. open surgery groups. (A) local recurrence and (B) distant recurrence rate. OR, odd ratio; LB, lower boundary; UB, upper boundary; CI, confidence interval.

Figure 4 presents the Forest plot of local recurrence rate (Fig. 4A), and distant recurrence rate (Fig. 4B). Five studies with complete data of local recurrence rates were included in the analysis. The heterogeneity test showed a fixed effect model was considered with a Q statistic = 4.10, and I^2^ = 2.43% (P = 0.393). The results with an OR = 2.152 (95% CI 1.349 to 3.434) indicated that VATS was associated with a higher local recurrence rate than open surgery, with a Z-statistic = 3.216 (P = 0.001; Fig. 4A).

Three studies with complete data of distant recurrence rates were included in the analysis of distant recurrence rate. The heterogeneity test shows a random effect model was considered with a Q statistic = 5.10 and I^2^ = 60.7% (P = 0.078). The results with an OR = 0.91 (95% CI 0.33 to 2.48) indicated no significant difference between the VATS and open surgery groups, with a Z-statistic = −0.18 (P = 0.856; Fig. 4B).

### Safety outcomes (rate of total complications, prolonged air leakage, arrhythmia, and pneumonia)

**Figure 5:**
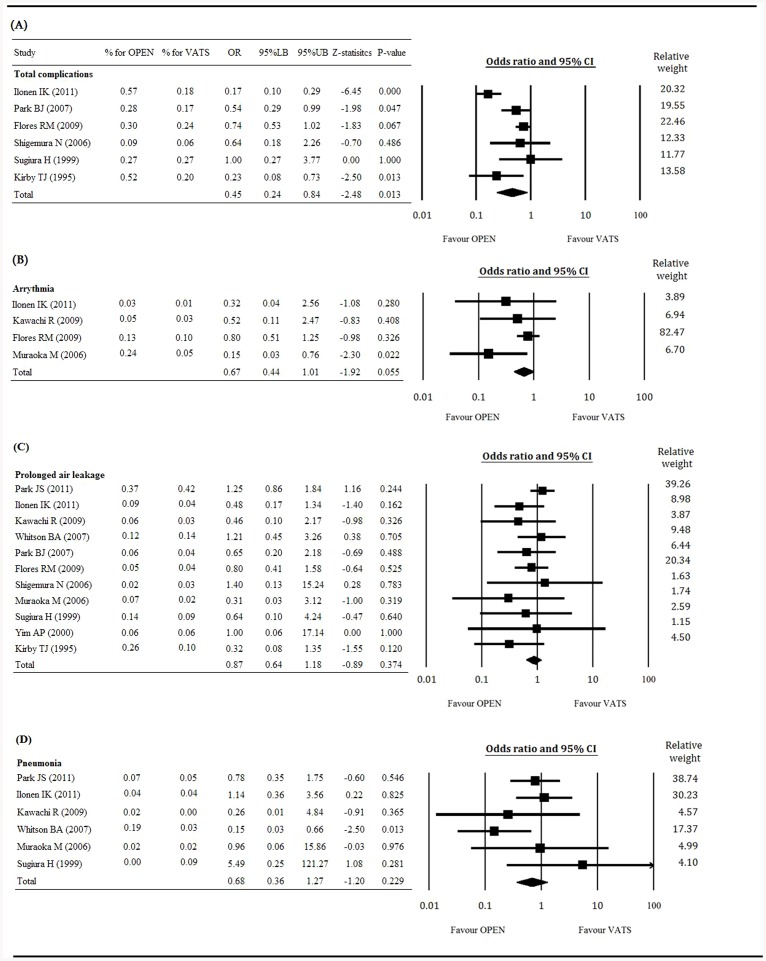
Forest plot for the VATS vs. open surgery groups. (A) total complications, (B) arrhythmias, (C) prolonged air leakage, and (D) pneumonia. OR, odd ratio; LB, lower boundary; UB, upper boundary; CI, confidence interval.

Figure 5 presents the Forest plots of total complications (Fig. 5A), arrhythmia (Fig. 5B), prolonged air leakage (Fig. 5C), and pneumonia (Fig. 5D). Six studies with complete data of the total complication rate were included in the analysis. The heterogeneity test shows a random effect model was considered with a Q statistic = 24.09 and I^2^ = 79.25% (P<0.001). The results with an OR = 0.45 (95% CI 0.24 to 0.84) indicated that VATS was associated with a lower rate of total complications than open surgery, with a Z-statistic = −2.48 (P = 0.013; Fig. 5A).

There were 4, 11, and 6 studies with complete data of arrhythmias, prolonged air leakage, and pneumonia, respectively. The results of the analyses indicated that VATS was associated with lower rates of arrhythmias (OR = 0.67, 95% CI 0.44 to 1.01, P = 0.055; Fig. 5B), prolonged air leakage (OR = 0.87, 95% CI 0.64 to 1.18, P = 0.374; Fig. 5C), and pneumonia (OR = 0.68, 95% CI 0.36 to 1.27, P = 0.229; Fig. 5D) than open surgery. However, there was not any statistically significant difference in these specific complications between the VATS and open lobectomy groups.

## Discussion

The results of this meta-analysis showed that VATS was associated with a better 5-year survival rate than open lobectomy in patients with stage I NSCLC, although VATS patients had a higher local recurrence rate than did those who received open surgery. Furthermore, there was no difference in distant recurrence rate between the 2 groups, while VATS was associated with lower rates of total complications, arrhythmias, prolonged air leakage, and pneumonia.

Six prior meta-analyses [5]–[9], [35] have examined VATS vs. open lobectomy in the treatment of early stage lung cancer (Table 2). While the disease stages and outcomes assessed are not exactly the same among the studies, the results of our analysis are generally in agreement with the prior studies which indicated that VATS is associated with a more favorable 5-year survival rate, either similar or lower rates of complications, and similar or lower rates of systemic recurrence as open lobectomy. In our study, however, VATS was not associated with a reduced local recurrence rate, which is different from the results of the other meta-analyses [5], [6], [8]. The inclusion and exclusion criteria of our study differed from those of the other meta-analyses, which may explain the disparate finding. Specifically, the meta-analyses reported by Yan et al [5] and Zhang et al [8] involved the results from studies of patients with early stage (stage I–IIIA) NSCLC, whereas the meta-analysis reported by Li et al [6] involved the results from studies of patients with stage I lung cancer (ie, not exclusively NSCLC).

**Table 2:** Comparison with prior meta-analyses.

Parameter	Current Study	Yan, 2009 [5]	Li, 2012 [6]	Cao, 2013 [7]	Zhang, 2013 [8]	Taioli, 2013 [9]	Chen, 2013 [35]
Disease type	Stage I NSCLC	Early-stage NSCLC	Stage I lung cancer	NSCLC*	Early-stage NSCLC	Lung cancer	Stage I NSCLC
Favor treatment	Favor VATS	Favor VATS	Favor VATS	–	Favor VATS	Favor VATS	Favor VATS
(Survival rate)	(5 year)	(5 year)	(5 year)			(5 year)	(5 year)
Length of hospitalization	-	-	-	VATS lower	-	-	VATS lower
Total LND or LNS procedures	-	-	-	-	No difference	-	-
Local recurrence	VATS higher	No difference	No difference	-	VATS lower	-	-
Systemic recurrence	No difference	VATS lower	VATS lower	-	VATS lower	-	-
Total complications	VATS lower	-	VATS lower	VATS lower	-	-	VATS lower
Prolonged air leakage	No difference	No difference	-	VATS lower	-	-	No difference
Arrhythmia	No difference	No difference	-	VATS lower	-	-	No difference
Pneumonia	No difference	No difference	-	VATS lower	-	-	VATS lower
Renal failure	-	-	-	VATS lower	-	-	-
Perioperative mortality	-	No difference	-	No difference	-	-	-

propensity score-matched.

In the Eastern Cooperative Oncology Group 3590 study, lymphadenectomy was defined as the removal of ≥10 lymph nodes from at least 2 or more mediastinal lymph nodes stations [36]. One of the chief concerns of VATS lobectomy is that it provides insufficient lymph node dissection. However, these concerns seem to be unjustified as studies have indicated that a standard lobectomy with lymph node dissection can be performed via VATS [37], [38]. Denliger et al. [39] reported that fewer lymph nodes were sampled with VATS lobectomy compared with open lobectomy; however, there was no survival difference. The authors believed that the reason fewer nodes were sampled with VATS lobectomy was because the subcarinal space does not have to be exposed in upper lobe lobectomies, thus dissection of the subcarinal lymph nodes is more challenging than that of other stations.

Our analysis showed that VATS was associated with a better the 5-year survival than open surgery, a result that is consistent with those of other meta-analyses and other studies [5], [6], [8], [9], [35]. Tahara et al. [36] reported that up to 25% of patients with T1 tumors had N+ disease at final postoperative pathological examination. However, studies have shown that patients who underwent VATS lobectomy who were stage N0 at clinical staging and who were found to have lymph node involvement at surgery or postoperative pathological examination have favorable outcomes [26], [40]. Kim et al. [40] reported that patients with pathological N1 or N2 disease after VATS lobectomy had a 3-year overall survival rate of 98% and 89%, respectively; rates comparable to that of open lobectomy. These data suggest that even in lymph node involvement is found during VATS lobectomy for clinical stage I disease, conversion to an open procedure is not necessary.

There are several potential explanations for the better 5-year survival with VATS compared with open surgery. One potential explanation is decreased release of cytokines with this approach, which would reduce the level of perioperative immunosuppression [41]–[43]. Another potential explanation is that patients who undergo VATS may be better able to tolerate postoperative chemotherapy [44], [45].

Our analysis indicated that VATS lobectomy for early stage NSCLC is associated with a lower rate of total complications, as well as lower rates of the individual complications of prolonged air leakage, arrhythmia, and pneumonia. These results are consistent with those of prior meta-analyses [5]–[9], [35].

Our meta-analysis has a number of limitations that must be considered when interpreting the findings described herein. The primary limitation is that the majority of the studies included were retrospective in nature. Another limitation is the fact that some of the studies included had a primary focus on safety or feasibility, rather than survival and/or recurrence. The methodologies employed in these studies may not have been appropriately rigorous regarding the assessment of survival and/or recurrence. We also acknowledge that various differences in between study factors that were not reported (eg, institutional VATS reliability, potential avoidance of central tumor location) may have affected the outcomes described and hence the results of our meta-analysis. Clearly, large-scale, multicenter, prospective studies would be warranted to account for these potential biasing factors.

### Conclusions

In summary, patients with stage I NSCLC undergoing VATS lobectomy had longer survival and fewer complications than those who received open lobectomy. These results suggest that VATS is an effective and safe approach for the treatment of early stage NSCLC.

## Supporting Information

Checklist S1 **PRISMA 2009 Checklist.**
(DOC)
